# Autism in the Faroe Islands: Diagnostic Stability from Childhood to Early Adult Life

**DOI:** 10.1155/2013/592371

**Published:** 2013-02-17

**Authors:** Eva Kočovská, Eva Billstedt, Asa Ellefsen, Hanna Kampmann, I. Carina Gillberg, Rannvá Biskupstø, Guðrið Andorsdóttir, Tormóður Stóra, Helen Minnis, Christopher Gillberg

**Affiliations:** ^1^Institute of Health and Wellbeing, College of Medical, Veterinary and Life Sciences, University of Glasgow, Caledonia House, Royal Hospital for Sick Children, Yorkhill, Glasgow G3 8SJ, UK; ^2^Gillberg Neuropsychiatry Centre, Institute of Neuroscience and Physiology, Sahlgrenska Academy, University of Gothenburg, Kungsgatan 12, 411 19 Gothenburg, Sweden; ^3^Department of Educational Psychology, Sernámsdepilin, Frælsinum 32, 100 Tórshavn, Faroe Islands; ^4^Child and Youth Psychiatry, Psychiatric Department, The National Hospital of the Faroe Islands, J. C. Svabosgøta, 100 Tórshavn, Faroe Islands; ^5^Genetic Biobank of the Faroes, Ministry of Health, J. C. Svabosgøta 43, 100 Tórshavn, Faroe Islands; ^6^Psychiatric Center, The National Hospital of the Faroe Islands, J. C. Svabosgøta, 100 Tórshavn, Faroe Islands

## Abstract

Childhood autism or autism spectrum disorder (ASD) has been regarded as one of the most stable diagnostic categories applied to young children with psychiatric/developmental disorders. The stability over time of a diagnosis of ASD is theoretically interesting and important for various diagnostic and clinical reasons. We studied the diagnostic stability of ASD from childhood to early adulthood in the Faroe Islands: a total school age population sample (8–17-year-olds) was screened and diagnostically assessed for AD in 2002 and 2009. This paper compares both independent clinical diagnosis and Diagnostic Interview for Social and Communication Disorders (DISCO) algorithm diagnosis at two time points, separated by seven years. The stability of clinical ASD diagnosis was perfect for AD, good for “atypical autism”/PDD-NOS, and less than perfect for Asperger syndrome (AS). Stability of the DISCO algorithm subcategory diagnoses was more variable but still good for AD. Both systems showed excellent stability over the seven-year period for “any ASD” diagnosis, although a number of clear cases had been missed at the original screening in 2002. The findings support the notion that subcategories of ASD should be collapsed into one overarching diagnostic entity with subgrouping achieved on other “non-autism” variables, such as IQ and language levels and overall adaptive functioning.

## 1. Introduction

Almost since the beginning of its history in clinical medicine, childhood autism/autistic disorder (AD) has been regarded as one of the most, if not the most, stable diagnostic categories applied to young children with psychiatric/developmental disorders [1, 2]. In the last several years, a number of studies have documented that autism is not a distinct “either/or” phenomenon, but often can be seen as a dimensionally distributed trait in the general population [3–5]. Recent hypotheses include those that see autism (or autism spectrum disorder (ASD), or pervasive developmental disorder (PDD)) as the lowermost portion on a spectrum of “autistic traits” shading into normally distributed similar traits in the population and that its basis is genetic, regardless of whether one is dealing with “caseness” or “the broader/normal phenotype” [6, 7]. However, the question remains as to whether, just as in intellectual developmental disorder (IDD), ASD as a clinical diagnosis, in some cases, represents pathological (and qualitatively different) variants that cannot be explained as a normally distributed trait (perhaps associated with brain damage or other non-genetic factors). Diagnostic stability would, hypothetically, be high for ASD under such a model. 

Papers reporting on diagnostic stability of ASD from 2005 onwards have concentrated on very young and preschool age children. Only one study reported a follow-up interval of 7 years (from age 2 through 9 years). Most studies compared the stability of clinical diagnosis over a 2-year period. The overarching category of ASD (encompassing all the diagnostic subcategories, including autistic disorder (AD), Asperger syndrome (AS), and PDD/not otherwise specified (NOS) [8, 9]) has repeatedly been reported as very stable (>90%), and the “core autism” (AD) and AS categories have been found to be more stable than the PDD-NOS category [10, 11]. Clinical diagnosis has consistently been shown to be more stable than any instrument diagnosis [12], such as diagnoses made using the Autism Diagnostic Interview-Revised (ADI-R) [13]; the Early Screening of Autistic Traits (ESAT) [14], Wing's [15] Autistic Disorder Interview (WADIC), and Autism Diagnostic Observation Schedule-Generic (ADOS-G) [16–18]; or the Childhood Autism Rating Scale (CARS) [19] and ADOS [17, 20].

A clinical diagnosis is usually considered the “gold standard.” However, for research purposes there has been a demand for some time for a “quantified” diagnostic measure and this has led to the development of some of the frequently used instruments: semi- or highly structured interviews (the ADI-R, or the Diagnostic Interview for Social and Communication Disorders (DISCO)), questionnaires (e.g., the Autism Spectrum Screening Questionnaire (ASSQ) [21–24], or the Social Communication Questionnaire (SCQ) [25]), and observation schedules (e.g., the ADOS [26]). There has also been a need to develop these scales for the purpose of training less experienced, junior clinicians or researchers to assist in the diagnostic process. This has led to the need for continuous research into diagnostic stability of ASD diagnoses made on the basis of different approaches (clinical “best estimate” or instrument diagnosis) and of compatibility across types of diagnosis made. It is essential that these instruments are compared with the clinical “gold standard.”

The stability over time of a diagnosis of ASD is not only theoretically interesting but important for a number of clinical reasons. Resources for psychoeducation and early intervention in ASD are currently allocated at a relatively high level in many western countries. The same holds for diagnostic services. Often, intervention provision is heavily dependent on availability of diagnostic services and knowledge about diagnostic stability, therefore, of particular importance.

We have had the opportunity to study the diagnostic stability of ASD from childhood to early adult life in a total population sample in the Faroe Islands. This paper details the results of that study, both as regards independent clinical comprehensive diagnosis and in respect of DISCO algorithm diagnosis of ASD at two time points, separated by seven years.

The Faroe Islands—considered a genetic isolate—are situated in the heart of the Gulf Stream in the North Atlantic Ocean, northwest of Scotland and half way between Norway and Iceland at 62°00′ N. It is a group of 18 islands, several of them now connected by under-sea tunnels. The total population of the Faroe Islands is about 49,000. There are only two towns—a capital Torshaven (around 19,000 inhabitants) and Klaksvik (around 5000). The rest of the population live in rural (including remote) areas and small villages.

Given the genetic isolate character, the Faroe Islands constitute an interesting environment in which to conduct epidemiological studies. Many variables are unusually stable, for example, socioeconomic status, education, health care, familial/genetic history, and diet among others. Several epidemiological studies so far have been interested in the apparently high prevalence of certain diseases in this community—among them Parkinson disease [27, 28] and autism [29].

## 2. Methods

In a general population setting in the Faroe Islands (in the North Atlantic Sea), we have been performing prevalence, clinical, and genetic studies of ASD for more than a decade. The school age child population as a whole (8–17-year-olds) on the islands was screened and diagnostically assessed for ASD in 2002 [29], and the same age cohort was screened and assessed again in 2009 [30].

### 2.1. Procedure

The same procedures for screening (ASSQ, school and hospital screening) and diagnostic assessments (clinical interview/assessment and Wechsler testing of the individual, DISCO interview with a parent) were employed at both time points. The ADOS was included only at Time 2. The clinician examining the individuals and interviewing the parents at Time 2 was usually not the same as had been involved at Time 1. Parents and teachers completed the ASSQ. Registers of schools and the Torshavn hospital (the only hospital in the Faroe Islands) were searched. Parents of screen positive individuals were interviewed using the DISCO-10. Screen positive individuals were themselves assessed using a semistructured interview, regarding their interests, skills patterns, family, and peer relationships. Their IQ levels were tested with the Wechsler Intelligence Scales for Children (WISC-R or WISC-III) [31, 32] or the Wechsler Adult Intelligence Scale-III [33] (in individuals over the age of 16 years).

### 2.2. Participants

The whole Faroese population of 8–17-year-old children (born in 1985 through 1994) was screened and diagnostically assessed for ASD throughout all schools and registers in 2002 (Time 1). The same age cohort was screened and assessed again in 2009 (Time 2). The details of the screening and diagnostic procedures included at Time 1 and Time 2 have already been published [29, 30]. Clinical interviews/assessments of the screen positive individuals were performed by one of two clinical psychologists (AE and HK) at Time 1 and by another psychologist (RB) (with no prior knowledge of the individuals and their diagnosis—this researcher was “blind”) at Time 2.

### 2.3. Instruments

*DISCO interviews* were done at both time points. They were performed by one of two clinical psychologists (AE and HK) at Time 1. DISCO-11 interviews were performed by a third clinical psychologist (RB) in the majority of cases (“old” and “new”) at Time 2. In 9 of the cases, for practical purposes, one of the two Faroese psychologists active at Time 1 performed the DISCO-11 interviews. In these cases, at Time 2, they each met a parent that they had interviewed personally at Time 1. The DISCO is an investigator-based structured and semistructured instrument developed with a view to serving as a research and clinical interview with a collateral informant (usually one of the parents, as in the present context) for differential diagnosis within the spectrum of autism and other social communication disorders [34, 35]. It has been used in a large number of studies (see Leekam [36] for a recent overview) and has been shown to have good to excellent psychometric properties including excellent interrater reliability and good validity for diagnoses within the autism spectrum [37]. It takes 2–4 hours to complete. It is currently available in its eleventh version (DISCO-11). The difference between the tenth (DISCO-10) and the eleventh version is minor. The DISCO-10 was used at Time 1 and the DISCO-11 at Time 2.

The DISCO provides a computerized diagnostic algorithm, allowing the following (mutually not exclusive) diagnoses to be made: “childhood autism/autistic disorder,” “atypical autism/PDD-NOS,” “Asperger syndrome according to ICD-10/DSM-IV, Asperger syndrome according to Gillberg” [38], “social impairment,” and “ASD” according to Wing [39]. Thus, the diagnosis is made by the computer on the basis of the clinical information given by the collateral informant and coded by the interviewer (AE and HB at Time 1 and in a few instances at Time 2, RB at Time 2) and is *not* at this “algorithm diagnostic stage” influenced by clinical comprehensive assessment, nor was the clinical diagnosis influenced by the DISCO algorithm diagnosis.

*Wechsler Intelligence Scales* were used age-appropriately for the cognitive assessment: WISC-III in the majority of cases at Time 1 and WAIS-R at Time 2. Those who were not tested at Time 1 were tested at Time 2. The Wechsler Intelligence Scale for Children is an individually administered intelligence test for children between the ages of 6–16 years. The WISC-R (Revised version) [31] and the WISC-III [32] has 15 subtests which are organized into verbal and performance scales and provide scores for Verbal IQ (VIQ), Performance IQ (PIQ), Processing Speed Index (PSI), and Full Scale IQ (FSIQ). Individuals over the age of 16 were tested with the Wechsler Adult Intelligence Scale (WAIS) [33].

*ADOS assessment* was performed only at Time 2. The ADOS is an instrument used for diagnosing and assessing autism. The protocol consists of a series of structured and semistructured tasks that involve social interaction between the examiner and the subject. The examiner observes and identifies segments of the subject's behaviour and assigns these to predetermined observational categories. Categorized observations are subsequently combined to produce quantitative scores for analysis. Research-determined cutoffs identify the potential diagnosis of autism or related ASD, allowing a standardized assessment of autistic symptoms.

### 2.4. Screening and Diagnosis Time 1

There were 56 children aged 8–17 years identified at screening with a suspicion of ASD from the population of 7,689 at Time 1 and 43 of these met DSM-IV PDD/ASD diagnostic criteria or, in the case of “Asperger syndrome,” they met criteria for this condition operationalised by Gillberg [38]. The parents of two of the 43 children did not wish for their child to participate in the in-depth assessment study but both these children had been worked up comprehensively and diagnosed with ASD by Faroese or Danish clinicians prior to the research study.

### 2.5. Screening and Diagnosis Time 2

All 41 participating individuals with ASD at Time 1, now aged 15–24 years, were contacted at Time 2 (2009) and 31 of these (76%) agreed to participate in the follow-up study. In addition, there were 30 individuals newly referred because of the suspicion of ASD during the 2009 screening, and 22 of these met diagnostic criteria for ASD. Two additional cases had received their clinical diagnosis elsewhere, but were confirmed by the Time 2 clinician (RB). This means that there were 55 cases available at Time 2 (31 + 22 + 2).

The reasons for refusal to participate in the study at Time 2 among the original group diagnosed at Time 1 included (as recorded by the DISCO interviewer) (a) parent's denial of any problems related to autism (*n* = 2); (b) autism individual's own denial of any problems (*n* = 2); (c) parent blaming the health system for not offering enough help (*n* = 2); (d) parents' refusal due to very low general functioning of the person with autism (*n* = 1); (e) involvement of genetic analysis in the study (*n* = 1); and (f) other “unspecified reasons” or “no information available” (*n* = 3).

### 2.6. Time 1 Final Sample

A total of 43 individuals received the diagnosis of ASD (autism, atypical autism, or Asperger syndrome) in 2002, corresponding to total population prevalence for ASD in 8–17-year-old children in the Faroe Islands of 0.56%.

### 2.7. Time 2 Final Sample

A total of 67 individuals received the diagnosis of ASD (autism, atypical autism/PDDNOS, or Asperger syndrome) in 2009, corresponding to total population prevalence for ASD in 15–24-year-old young adults in the Faroe Islands of 0.94% [30]. Of these, 24 were “new cases,” not found in the study at Time 1. For these, of course, there were no Time 1 assessments, clinical or DISCO algorithm ASD diagnoses available. Only in those individuals who had been assessed both at Time 1 and Time 2 was it possible to study diagnostic stability over time (*n* = 31 for clinical diagnosis, *n* = 30 for DISCO algorithm diagnosis).

The source of referral to the study for ASD diagnostic assessment after screening of the 24 new cases at Time 2, who were missed at Time 1, included (a) the Torshavn Hospital Adolescent Psychiatry Outpatient Unit where they had been treated for other mental health problems: anxiety (*n* = 2), depression (*n* = 2), ADHD (*n* = 2), and other (*n* = 5); (b) adult psychiatrists who had treated them as outpatients for other mental health problems: depression (*n* = 1), psychosis (*n* = 1), and other (*n* = 6); or (c) the Torshavn Hospital Adult Psychiatry Inpatient Unit where they were treated for other psychiatric disorders: depression (*n* = 4) and OCD (*n* = 1).

The mean age of the DISCO algorithm diagnostic stability study group at followup was 19.5 (SD 3.1) years. There were 5 females (17%) and 25 males (83%). IQ subcategories were defined as follows: IQ 20–50, *n* = 9 (30%); 51–70, *n* = 3 (10%); 71–85, *n* = 7 (23%); >85, *n* = 11 (37%). 

Eleven of the Time 1 sample and 6 of the Time 2 sample of individuals failed to take part in the DISCO-11 assessment, leaving 50 probands at Time 2 for whom there was both a DISCO-11 algorithm diagnosis and an independent clinical diagnosis (including 20 for whom there was no Time 1 diagnosis).

### 2.8. Statistical Analysis

All statistics were calculated via SPSS 17.0 software on anonymous data, using two-tailed *P* values. *P* values < 0.05 were considered statistically significant. An agreement between diagnostic raters at two time points in 2002 and 2009 was quantified by using Kappa statistics. Kappa score was assigned according to Landis and Koch scale [40] using 95% confidence intervals.

### 2.9. Ethics

The study was approved by the Faroe Islands Board for Ethics in Medicine. All families provided informed consent (parents or, in the case of individuals 18 years or over, by the individuals with a diagnosis of ASD (from Time 1) or with suspected autism spectrum problems (from Time 2) themselves).

## 3. Results

### 3.1. Stability of Clinical Diagnosis

When combining the AD, AS, and atypical autism/PDDNOS into a collapsed ASD group, 30 of 31 (97%) remained in this overarching clinical diagnostic category. When separating them into specific ASD diagnostic subcategories (Table 1), those with an AD diagnosis in 2002 (*n* = 10) all maintained their diagnosis, whereas in the group with an original diagnosis of AS in 2002 (*n* = 15), 5 were no longer diagnosed in this category (4 with atypical autism/PDDNOS and 1 with no ASD diagnosis at all at Time 2). All but one of those with an atypical autism diagnosis in 2002 (*n* = 6) were still diagnosed in this category at followup (one male in this subgroup was diagnosed with AS at Time 2).

An agreement between clinical diagnoses in 2002 and 2009 was quantified by using Kappa statistics. Kappa score was 0.747 (95% confidence interval: from 0.548 to 0.945). The strength of agreement is considered to be “good” (substantial).

### 3.2. Clinical Subgroup Characteristics according to Change/No Change of Diagnostic Category

The 6 individuals (5 males) whose original clinical diagnosis of AS or atypical autism/PDDNOS had changed at Time 2 had a mean age at followup of 21.0 (SD 3.6) years; their IQ ranged from 50 to 102; 5 individuals had low scores (0-1) on the ADOS “Stereotypical/Repetitive Behaviour” scale. This was markedly different from the group of 10 (8 males) individuals with an original clinical diagnosis of AD (all of whom were again diagnosed clinically as AD at Time 2). Their mean age was 19.3 (SD 3.6) years (n.s.), all but 1 had IQ < 50 (*P* < .001), and all had ADOS Stereotypical/Repetitive Behaviour scores of 2 or more (*P* < .01). However, the AS/atypical autism/PDDNOS group that remained stable (*n* = 15, 12 males) did not differ from those that changed, in terms of ADOS Stereotypical/Repetitive Behaviour scores, but they were younger at followup (18.7, SD 2.8 years, *P* < .05) and IQ tended to be a bit higher (range 73–114).

### 3.3. Stability of DISCO Algorithm Diagnosis

Of all five DISCO algorithm diagnoses, the category of AD was the most stable between 2002 and 2009 (8 of the 10 individuals remained in the same category) (Table 2). The DISCO algorithm diagnoses of AS and atypical autism showed considerable variability; however, no individual moved out of the overarching ASD category altogether.

An agreement between DISCO algorithm diagnoses in 2002 and 2009 was quantified by using Kappa statistics. Kappa score was 0.299 (95% confidence interval: from 0.099 to 0.500). The strength of agreement is considered to be “fair.”

### 3.4. Correspondence between Clinical Diagnosis and DISCO Diagnosis at Followup (*n* = 50)

The highest agreement/stability between the Clinical ICD-10/DSM-IV diagnosis and DISCO algorithm diagnosis in 2009 was noted for AD (67% complete agreement) and AS (52% complete agreement) (Table 3).

An agreement between clinical ICD-10 diagnosis and DISCO diagnosis in 2009 was quantified by using Kappa statistics. Kappa score was 0.502 (95% confidence interval: from 0.278 to 0.726). The strength of agreement is considered to be “moderate.”

### 3.5. Gender Effects

Among the females (*n* = 6) who participated in the follow-up study, 5 remained in the original diagnostic category whereas 1 woman, earlier diagnosed with Asperger syndrome, now received the diagnosis of atypical autism.

There were more females identified at Time 2 (*n* = 11 ~ 45.8%): 1 with childhood autism, 8 with Asperger syndrome, and 2 with atypical autism diagnosis, in comparison to the original study at Time 1 (*n* = 7 ~ 16.3%): 4 with childhood autism and 3 with Asperger syndrome diagnosis, indicating that more females were missed at younger ages.

## 4. Discussion

Interestingly, the stability of clinical ASD diagnoses was perfect for AD, good for atypical autism/PDD-NOS, and less than perfect for AS. Stability of the DISCO algorithm subcategory diagnoses was more variable but still good for AD. In terms of “any ASD” diagnosis, both systems showed excellent stability over the seven-year period with only one case of “clinical ASD” at Time 1 receiving “no clinical diagnosis” at Time 2 and one case of “No DISCO ASD-diagnosis” at Time 1 receiving a “DISCO-ASD diagnosis” (AS) at Time 2.

Before going on to discuss the implications of the findings, several things need to be addressed. First, what is the representativeness of the sample? Even though relatively small, the groups studied are representative of the total population of young people with ASD in the Faroe Islands, as has been argued in more detail in a previous publication by our group [30]. The fact that they were recruited in a genetic isolate could, by some, be taken to indicate that they might be atypical, and findings therefore not generalisable to other populations. Even though this cannot be absolutely excluded, several members of the research group have experience of working with thousands of individuals with ASD, and their conclusion is that the Faroe Islands ASD groups are typical of similar age groups with ASD in other countries.

Second, was the clinical diagnostic process sufficiently expert and in-depth to allow generation of valid comprehensive clinical ASD diagnoses? We would argue that indeed it was. The individuals in the study were examined for many hours, and on several different occasions, by experienced psychologists and psychiatrists. These experts were working in the context of an internationally well-known and clinically highly experienced research group, who has demonstrated excellent reliability for autism diagnoses [41]. 

Third, is the DISCO an instrument with established psychometric properties? The DISCO has excellent inter- and (short-term) intrarater reliability and is valid for ASD diagnoses, both as derived from clinical assessment and after interview using an alternative investigator-based collateral informant interview, the ADI-R [37]. The DISCO generates much more information about early development and ASD-associated (not just “ASD-diagnostic”) symptoms and problems, and so it is our contention that it is at least as useful in ASD diagnostics as is the ADI-R.

Finally, were the diagnosticians independent of each other and in relation to the DISCO algorithm diagnoses when they made their clinical diagnosis within the spectrum of autism? All clinical diagnoses were made on the basis of all available information obtained by each Faroese clinician (sometimes with the help of the Swedish clinical researchers) without any knowledge of the DISCO algorithm ASD diagnoses delivered by the computer. It could not be ruled out that information obtained at DISCO interview might have influenced the Faroese clinician when assigning a clinical diagnosis, but the algorithm diagnosis (a complex combination of a very large number of items from all the many areas covered by the DISCO) and its constituent parts were not known to the clinician when the diagnosis was made. On balance, therefore, we conclude that the findings obtained are highly relevant as a basis for discussion of the stability and interrelationship of clinical and DISCO diagnoses of ASD in a long-term perspective.

There were no significant gender effects as regards stability/change of diagnosis, either in respect of clinical or DISCO algorithm diagnoses. However, the number of female cases included in the study was low (even though several previously undiscovered cases were identified at the second study), meaning that conclusions can only be tentative in this respect. In effect, one might argue that the relatively high number of “new” female cases emerging at Time 2 could be seen as an indication of the poor “diagnostic stability” of ASD in females (noncaseness turning into caseness at a considerable rate over a seven-year period, in spite of the “true” onset of the ASD having been in early childhood in all the “new” female cases).

It appears, then, that the take home message from this study is that both clinical and DISCO algorithm diagnoses are stable over the period from school age through late adolescence and early adult life so long as one is referring to ASD and not to individual categories within the ASD umbrella concept. For autistic disorder/childhood autism the clinical diagnosis is very stable, and the DISCO algorithm diagnosis fairly stable over a 7-year period from school age to early adult life. Asperger syndrome “caseness,” on the other hand had relatively poor predictive ability for the same diagnosis at Time 2, with a “hit rate” of 67% for clinical and only 27% for DISCO algorithm identical diagnosis at followup.

In summary, the results of this study could be taken to lend support for the notion that a single diagnostic category, “autism,” or “ASD” would be better suited to clinical realities than the current subdivision into autistic disorder, Asperger syndrome, childhood, and PDDNOS/atypical autism. At the time of writing, a single autism diagnostic entity is what is being proposed by the DSM-5 committee for neurodevelopmental disorders (and most likely the corresponding ICD-11 committee also).

## Figures and Tables

**Table 1:** Stability of clinical diagnosis from 2002 to 2009 (*n* = 30).

Clinical diagnosis 2002	Clinical diagnosis 2009			
	Atypical autism	Asperger syndrome	AD	Total
Atypical autism	5	1	0	6
Asperger syndrome	4	10	0	14
AD	0	0	10	10

Total	9	11	10	30

Kappa score: 0.747 ~ good (95% ci: 0.548–0.945).

**Table 2:** Stability of DISCO algorithm diagnosis from 2002 to 2009 (*n* = 30).

DISCO algorithm diagnosis 2002	DISCO algorithm diagnosis 2009					
	SID*	ASD**	Atypical autism	Asperger syndrome***	AD	Total
SID*	1	0	1	0	0	2
ASD**	0	0	0	0	0	0
Atypical autism	2	0	1	2	0	5
Asperger syndrome***	2	3	2	4	2	13
AD	1	0	1	0	8	10

Total	6	3	5	6	10	30

*Social interaction disorder according to Wing and Gould criteria.

**Autism spectrum disorder according to Wing and Gould criteria.

***Asperger syndrome according to Gillberg and Gillberg criteria.

Kappa score: 0.299 ~ fair (95% ci: 0.099–0.500).

**Table 3:** Correspondence between clinical diagnosis and DISCO diagnosis at followup (*n* = 50).

Clinical ICD-10 diagnosis 2009	DISCO diagnosis 2009			
	Atypical autism	Asperger syndrome	AD	Total
Atypical autism	5	1	0	6
Asperger syndrome	5	12	2	19
AD	2	2	8	12

Total	12	15	10	37

Kappa score: 0.502 ~ moderate (95% ci: 0.278–0.726).
